# Metagenomic and proteomic analyses reveal similar reproductive microbial profiles and shared functional pathways in uterine immune regulation in mares and jennies

**DOI:** 10.1371/journal.pone.0321389

**Published:** 2025-04-16

**Authors:** Eva da Silva-Álvarez, Vanessa Gómez-Arrones, Florencia Correa-Fiz, Francisco Eduardo Martín-Cano, Gemma Gaitskell-Phillips, Juan Jesús Carrasco, Joaquín Rey, Inés María Aparicio, Fernando Juan Peña, Juan Manuel Alonso, Cristina Ortega-Ferrusola

**Affiliations:** 1 Department of Animal Medicine, Laboratory of Equine Reproduction and Equine Spermatology, Faculty of Veterinary Medicine, University of Extremadura, Cáceres, Spain; 2 Centro de Selección y Reproducción animal de Extremadura. Junta de Extremadura, Badajoz, Spain; 3 Centre de Recerca en Sanitat Animal (CReSA), Unitat Mixta d’Investigació IRTA-UAB en Sanitat Animal, Campus de la Universitat Autònoma de Barcelona (UAB), Barcelona, Spain; 4 IRTA, Programa de Sanitat Animal, Centre de Recerca en Sanitat Animal (CReSA), Campus de la Universitat Autònoma de Barcelona (UAB), Barcelona, Spain; 5 WOAH Collaborating Centre for the Research and Control of Emerging and Re-Emerging Swine Diseases in Europe (IRTA-CReSA), Barcelona, Spain; 6 Department of Animal Health, Unit of Infectious Diseases, University of Extremadura, Caceres, Spain; 7 Department of Anatomy, Cell Biology and Zoology, Faculty of Nursery and Occupational Therapy, University of Extremadura, Caceres, Spain.; Yantai Institute of Technology, CHINA

## Abstract

This study aims to unveil potential differences in the vaginal and uterine microbiomes in mares and jennies, and to identify possible mechanisms involved in uterine immune homeostasis. The microbiota was characterized using 16S rRNA sequencing, and the uterine proteome was analyzed using UHPLC/MS/MS in 18 samples from healthy mares and 14 from jennies. While taxonomic analysis revealed high interspecies similarities, β-diversity analysis showed distinct clustering, with only two vaginal taxa and five uterine taxa differing between species. Despite compositional differences, PICRUSt analysis suggested minimal variations in predicted functional pathways across species. Comparing vaginal and uterine microbiota within the same species revealed overlapping bacterial taxa, but significant differences in α- and β-diversity and functional pathways. The uterine microbiota of both species was dominated by Proteobacteria, Firmicutes, Bacteroidetes, and Actinobacteria, with abundant taxa like *Streptococcus, Pseudomonas*, Bacillus, Corynebacterium, and Staphylococcus, many of which are frequently associated with endometritis. The presence of *Lactobacillus* in the equine reproductive tract was minimal or non-existent. KEGG functional pathway analysis predicted that uterine microbiota of both species utilize metabolic pathways with potential immunomodulatory effects. Proteomic enrichment analysis showed that numerous overexpressed uterine proteins in both species are linked to adaptive and innate immune regulation and defense mechanisms against symbionts. Gene enrichment analysis identified several enriched Gene Ontology terms, including response to bacterial stimuli, humoral immune regulation, and TGF-beta receptor signaling, underscoring microbial-host interactions. The uterine microbiota may play a vital role in maintaining immune balance. Further research is required to confirm its interaction with the uterine immune system and clarify the mechanisms involved.

## Introduction

Recent advancements in omics technologies have enhanced our understanding of the uterine environment and pathogenesis of uterine disorders such as endometritis in equines [1–3]. Equine persistent endometritis is considered one of the main causes of subfertility in mares *(Equus ferus caballus)* and jennies *(Equus africanus asinus)*, causing significant economic losses in the equine industry [4]. Endometritis is caused by several predisposing factors (mechanical, immunological and microbial) that can disrupt the uterine environment, often leading to infection. The primary challenge in infectious endometritis is that its treatment typically requires antibiotic administration. Development of probiotics is generating significant interest due to the need to replace antibiotics with alternative solutions. However, their development requires identification of probiotic bacteria present in a balanced ecosystem. The identification of microorganisms has traditionally been performed using culture-dependent methods [5]. However, microbial culture only detects and quantifies bacteria capable of growing under selective conditions, leading to an underestimation of bacterial diversity [6]. Additionally, the upper genital tract, including the uterus, is characterized by a low-biomass microbiota, which makes standard culture-based techniques inadequate for identifying the resident microbial community [6,7]. Advanced bacterial identification methods, such as 16S rRNA gene sequencing, have shown that the reproductive tract in various species harbors a complex and dynamic microbial ecosystem [8]. Moreover, shifts in uterine and vaginal microbial diversity and composition have been associated with pregnancy loss and negative obstetric outcomes in women [9–12]. The vaginal microbiome has been extensively studied in humans, where *Lactobacillus* is recognized as the dominant genus in healthy vaginal flora, playing a crucial role in preventing opportunist pathogen proliferation by maintaining an acidic vaginal pH [13]. Indeed, Lactobacilli probiotics are already being implemented as a therapeutic strategy for vaginal bacterial dysbiosis in women [14,15].

Recently the vaginal and uterine microbiota has been sequenced in several animal species, including cows, giant pandas, ewes, mares or jennies [1,16–21]. Research suggests that the uterine microbiome is unique to each species [8,22,23] and that its composition may be affected by anatomical locations or niches within the same species [7,20]. Besides, differences in the equine uterine microbiota have been attributed to various factors, including hormonal fluctuations, geographical location, or health status [1,2,11,24]. The female horse (mare) and donkey (jenny) are two subspecies of equids that share numerous reproductive aspects due to their phylogenetic proximity. Investigating whether both species share a similar resident uterine microbiome at both compositional and functional levels could provide new lines of research for the development of innovative therapeutic strategies. A deeper understanding of the uterine microbiota and its interactions with the immune system could significantly advance our knowledge of the pathophysiology of endometritis and equine fertility. Although shifts in microbiome diversity have been linked to endometritis in both species [1,2], the role of uterine microbiota and the specific mechanisms by which these microbiotas communicate with the uterine immune system remain poorly understood. Evidence suggests that the uterus may harbor a unique low-biomass microbiota capable of modulating inflammation and promoting immunotolerance, but further research is essential to elucidate these interactions and their potential therapeutic applications.

In recent years, the equine uterine proteome has been studied with different proposes, offering significant advancements in understanding the molecular mechanisms underlying reproductive physiopathology [25–27]. These studies have identified potential biomarkers for the diagnosis of endometritis and provided valuable insights into novel therapeutic strategies [3,28]. Therefore, the characterization of the uterine microbiota in conjunction with proteomic analysis of uterine fluid could improve our understanding of the complex interactions between the host immune system and the microbiota within a healthy uterine environment. The objectives of the current study were (i) to compare the vaginal and uterine microbiome and proteome of healthy mares and jennies; (ii) to provide a comprehensive understanding of how these microbiotas contribute to homeostasis with the uterine immune system and explore the potential roles of uterine proteins in the regulation on endometrial immunology.

## Materials and methods

### Study design

In this study, the resident vaginal and uterine microbiomes of healthy mares and jennies were characterized using 16S rRNA sequencing of vaginal swabs and uterine lavage fluid. Additionally, the analysis of the uterine fluid proteome was performed using UHPLC/MS/MS. Paired samples of vaginal swabs (cranial vagina) (VS) and uterine fluid (UF) were obtained from 9 mares and 7 jennies in two consecutive follicular phases. The lavage fluid was divided under sterile conditions into four 50-mL Falcon tubes, designated for cytology, culture, 16S rRNA sequencing, and proteomic analysis. The samples intended for metagenomic and proteomic analysis were kept at −80°C until they were processed. A total of 64 samples were analyzed: 36 samples from mares (18 vaginal samples and 18 uterine samples) and 28 samples from jennies (14 vaginal samples and 14 uterine samples).

### Animals

Mares were kept according to institutional and European animal care regulations (Law 6/2913 June 11 and European Directive 2010/63/EU), and all experimental procedures were reviewed and approved by the Ethics committee of the University of Extremadura, Caceres, Spain. Jennies belonging to CENSYRA (Centro de Selección y Reproducción Animal) were also kept according to institutional and European animal care regulations. All animals were kept in paddocks with access to ad libitum water and fed grass hay in addition to grazing. A total of 9 healthy Pure Spanish Horse mares (11–14 years old) and 7 Andalusian donkey jennies (5–16 years old) were used in this study. The mares were sampled at the Clinical Veterinary Hospital (CVH) of the University of Extremadura, located in Cáceres (northern Extremadura). The mares came from two nearby farms in Cáceres, where they were born and raised.

The jennies were sampled at CENSYRA (Centro de Selección y Reproducción Animal) in Badajoz (southern Extremadura). All the jennies were born and raised at the center and are breeding animals that are inseminated annually with different stallions. Four of the mares were maidens, and the rest of the females were multiparous. A complete reproductive examination, including transrectal palpation, ultrasonographical monitoring (B-mode and color Doppler), and endometrial cytology and culture was performed in all females. All animals enrolled in this study cycled regularly, and none of them had clinical signs of endometritis.

### Vaginal and uterine sampling procedure

Vaginal and endometrial samples were collected during the follicular phase under strict hygiene measures to prevent contamination. Sampling criteria included a follicle ≥ 35 mm in diameter, endometrial edema (score ≥ 3/5), a relaxed cervix, and a positive teasing response. The procedure involved wrapping and bandaging the tail, washing the vulva and perineum three times with pH-neutral soap and water, and carefully inserting a sterile bi-valve speculum into the vaginal cavity to obtain cranial vaginal exudate using a sterile double-guarded culture swab (Minitube-Iberica, Spain). Samples were obtained by gentle swabbing of the vaginal wall at the level of the fornix and around the cervix (cranial vagina) for 30 s. The swab was immediately placed in transport tubes, which were sealed and protected from light. The swab was frozen at −80°C for metagenomic analysis.

For endometrial samples, a uterine flushing catheter (IMV, Spain) connected to a Foley catheter and a 500mL bottle of sterile 0.9% sodium chloride solution warmed to 37°C was used. All material was sterile, and the circuit was prepared in an adjacent laboratory under a laminar flow chamber to maintain maximum sterility. The catheter was protected with a transrectal glove during its passage through the vagina and it was inserted through the cervix. The fluid was infused using gravity and left inside the uterus for 2 minutes. Immediately, before the fluid was recovered, the uterus was massaged per rectum to ensure the fluid was evenly distributed to obtain a more representative sample of the entire uterus. The fluid was then recovered using gravity, lowering the fluid bottle to below the level of the uterus, assisted by transrectal uterine massage. The sample obtained via uterine flushing was aspirated from the bottle using a sterile syringe under a laminar flow chamber and divided among 4 sterile falcon tubes, which were sealed and protected from light. One was sent immediately for culture-dependent bacteriological identification, another was processed for cytology and the remaining tubes were frozen at −80°C for posterior metagenomic and proteomics analysis.

### Cytological endometrial analysis

Endometrial cytology was performed on fluid recovered from uterine lavage. To do this, the 50 ml falcon tube was centrifuged at 400g for 10 minutes, and samples were prepared according to protocol described by Bohn et al. [29]. Samples were assessed at 400x magnification and inflammatory cells were identified and counted in ten different microscopic fields. Cytological smears were graded as not inflammatory (< 2 neutrophils/field), moderate inflammation (2–5 neutrophils/field), severe inflammation (>5 neutrophils/field)[30].

### Uterine bacteriological analysis (culture-dependent processing)

Samples from uterine lavage were sent to the Infectious Diseases Laboratory at the University of Extremadura for isolation and identification using conventional culture techniques, as described in Da Silva et al., 2024 [3].

### Sample collection for 16s RNA sequencing

Uterine fluid and vaginal swabs from the cranial vagina were kept stored at −80ºC and sent to the Applied Bioscience Techniques Service (STAB) at the Central Services of the University of Extremadura.

### Total DNA isolation from uterine and vaginal samples

For metagenomic analysis, samples (50 ml sterile tube) were thawed on ice. Samples were centrifuged at 1800g for 15 min at 4ºC. The supernatant was removed, and the precipitate was re suspended in 200 µl of sterile PBS, which was previously filtered through 0.22 µm. For analysis of vaginal samples (VS), 500 µl of sterile PBS was added and then filtered through 0.22um and vortexed for 3 min. Samples were then rested for a further 10 min at room temperature and 200 µl of the medium separated. From this step onwards, the samples obtained by uterine lavage and those from swabs were processed in the same way, using the MagMax Pathogen RNA/DNA kit (Thermo Fisher Scientific, USA) following the manufacturer’s instructions and using the automatic KingFisher Flex (Thermo Fisher Scientific, USA) nucleic acid extractor.

### 16S library preparation for NGS

The Ion 16S™ Metagenomics Kit (Thermo Fisher Scientific, USA) was used for the analysis of variable bacterial populations from vaginal and uterine samples. For this, the Ion Torrent™ (Thermo Fisher Scientific, USA) semiconductor sequencing workflow technology using established protocols for amplicon, library, and template preparation was used. The kit allows the amplification by polymerase chain reaction (PCR) of hypervariable regions of 16S rDNA from bacteria. Following this, the amplified fragments can be sequenced using the Ion PGM™ 400 sequencing kit on the Ion PGM™ System (Thermo Fisher Scientific, USA), in addition to analysis of the results using the Ion Reporter™ software Ion 16S™ Metagenomics Kit analysis module. Once the genomic DNA from all the samples had been extracted, a library of 16S rRNA/DNA fragments present in each one of them was prepared and the Ion AmpliSeq Microbiome Health Research kit (Thermo Fisher) was used. This kit consists of a panel of two pools of primers and the necessary reagents to generate the amplicons that make up the NGS library for the Ion Torrent platform. The kit includes two sets of primers that can be used to amplify the corresponding hypervariable regions of the 16S rDNA gene in bacteria: Primer set V2-4–8*,* Primer set V3-6, 7–9. These complete primer sets allow for precise detection and identification of a wide range of bacteria up to the genus or species level within a mixed population. Both primer sets are accompanied by a v2.0 Environmental Master Mix, which has been optimized to tolerate high levels of PCR inhibitors and to amplify targets in complex samples, such as environmental, food and tissue samples. Of the two pools only one of them was used, which contains 8 of the 9 hypervariable regions of the 16S rRNA coding gene. The protocol followed for the preparation of the library was that specified by the manufacturers, which included the quality control points for the samples during the process. In addition to the samples analyzed, two control samples were included; one containing genomic DNA from *E. coli* as a positive control and the other in which no genetic material was added as a negative control. The different libraries were indexed with a barcode (IonCode Barcode Adapters 0101–0196) included in the kit. Once preparation of the libraries was complete, they were quantified by QPCR with the Ion Universal Library Quantitation kit (Thermo Fisher) following the manufacturer’s instructions. Once the concentration of each was identified, they were diluted to 10 pM and an equimolar mixture was formulated for loading at a later stage on an Ion 530 chip. Chip loading was done using the Ion Chef with the reagents from the Ion 510 & Ion 520 & Ion 530 Kit-Chef kit (Thermo Fisher). Once the chip had been prepared, the libraries were sequenced using the Ion S5 Xl available at the Applied Bioscience Techniques Service (UEx) with the reagents included in the aforementioned kit.

### Bioinformatic analysis of 16s rRNA data

Raw data was obtained directly from the sequencer as fastq files containing the different amplicons from each sample. The downstream analysis was mainly done using the Quantitative insights into microbial ecology software [31] (Qiime2 vs 2023.7). For both denoising and quality-control, the q2-dada2 plugin was used over each run independently using the denoise-single function using no truncation and *p-trim-left* 15[32]. Additionally, Amplicon Sequence Variants (ASVs) obtained from dada2 that did not align with the Greengenes database version 13.8 at a minimum of 88% preclustering, 65% identity [33], and 50% query coverage were excluded using the VSEARCH tool within the q2 quality-control plugin[34]. This step allows for elimination of spurious non-prokaryotic elements considered unspecific contaminants. Curated merged sequences were aligned with sepp [35] to address the issue of proprietary primers preventing variable-specific amplicon deconvolution. Hypervariable positions were masked [36] with q2 alignment plugin, and the phylogenetic tree was built using Fastree [37]. Each amplicon sequence variant was assigned through the Qiime2 classifier, using the *q2-feature-classifier plugin* using full-length 16S Greengenes classifier (available at https://docs.qiime2.org/2022.2/data-resources/) and VSEARCH algorithm [37]. Sequences identified as non-bacterial, such as those belonging to the *Archaea* domain, Chloroplast, or Mitochondria, were also filtered out from the dataset together with those taxa found in the negative controls.

For the diversity analysis, the rarefaction plots were explored to determine the minimum depth for use in the *core-metrics* plugin. Phylogenetic methods were preferred to analyze the different amplicons collapsed by phylogeny, avoiding exaggeration of diversity estimates due to distinct amplicons by splicing in the same branch of the tree. Diversity analyses were performed at a common depth of 1,300 for the uterine and 5,800 for vaginal samples, corresponding to the lowest sample depth. Alpha diversity measures were conducted employing Faith's Phylogenetic Diversity (PD-Faith) diversity index [38], a measure of biodiversity that takes into account the phylogenetic distance between species in a community, calculating the sum of the phylogenetic branch lengths connecting all species present in a sample. Richness was estimated through the Chao index [39]. The significance of the differences between the study groups were tested with pairwise t-tests (999 random permutations using *alpha-group-significance* [40]. For beta diversity, distance matrices were derived using the Unifrac indexes [41] (weighted and unweighted). To determine whether the centroids of two or more groups differed significantly, a permutation-based analysis of variance (PERMANOVA) was employed, included in *beta-group-significance* [42]. Additionally, the proportion of variance accounted for by the study groups was determined using the Adonis function within the Vegan package in R [43]. To perform differential analysis, the analysis of composition of microbiomes with a bias correction (ANCOM-BC) [44] algorithm was done collapsed at different taxonomic levels to avoid bias generated by the fact that different regions from the 16S rRNA gene are included in the analysis. Picrust2 analysis was used for the functional prediction from the inferred metagenome. All singletons were removed and EPA-NG [45] was used for the phylogenetic placement of reads and KEGG orthologs database to infer gene families and functional pathways. STAMP was used for computing statistics and for visualization [46]. Plots and figs were built using R studio version 2022.07.0 [47] and qiime2R, ggplot2 [48] and tidy verse packages.

### Uterine proteomics analysis

#### Sample concentration.

Proteomic analysis included 32 samples: 18 uterine samples from mares and 14 uterine samples from jennies, with two technical replicates for each. Due to the low protein concentration in the uterine lavages, the samples were concentrated using 5000 Da cut-off 20 mL centrifugal concentrators (Spin-X UF 20, Corning, Germany). In short, 20 mL of uterine lavage was added to the top of the filter and centrifuged at 4000g for 2 hours at 4°C. After centrifugation, the concentrate was transferred to a cryovial and stored at −80°C until further analysis.

#### Protein quantification.

Protein quantification was conducted using the 2-D Quant Kit (GE Healthcare, Sevilla, Spain) by following the provided instructions: https://www.gelifesciences.co.jp/tech_support/manual/pdf(806486. From each sample, 100 μg of protein was measured based on the concentration results of protein quantification.

#### In-solution trypsin digestion.

Due to varying protein concentrations among the samples, Type 1 water was added to each to achieve a uniform starting volume of 300 μL. Subsequently, 25 μL of 0.1 M ammonium bicarbonate buffer (pH 8.5) containing 0.01% Surfactant (ProteaseMAX, Promega, USA) was added. The proteins in this solution were reduced by adding 1.5 μL of 200 mM dithiothreitol (DTT) and incubating at 56°C for 20 minutes. Then, the proteins were alkylated by adding 3 μL of 200 mM iodoacetamide (IAA) and incubating for 45 minutes at room temperature in the dark. To neutralize any excess IAA, samples were further incubated at room temperature in the dark for 30 minutes after adding 1 μL of 200 mM DTT. Protein digestion was then carried out by adding 2 μL of Trypsin Proteomics Grade (Sigma) (Trypsin solution: 1 μg/μL in 1 mM HCl) and incubating overnight at 37°C. The reaction was terminated by adding 1 μL of 99% formic acid and centrifuging for 5 minutes at 7000g. The supernatant was transferred to a new 1.5-mL vial. Finally, samples were dried using a speedvac (GYROZEN; 1 bar, 2000 rpm, 3 hours, 35°C). The dried samples were re-suspended in 20 μL of mass spectrometer mobile phase, composed of water/acetonitrile/formic acid (94.9:5:0.1).

#### UHPLC–MS/MS analysis.

The separation and analysis of the samples were conducted using a UHPLC-MS/MS system, specifically an Agilent 1290 Infinity II Series UHPLC (Agilent Technologies, Santa Clara, CA, USA) equipped with an automated multisampler module and a high-speed binary pump. This setup was connected to an Agilent 6550 Q-TOF Mass Spectrometer (Agilent Technologies) using an Agilent Jet Stream Dual electrospray (AJSDual ESI) interface. The UHPLC and Q-TOF were controlled via MassHunter Workstation Data Acquisition software (Agilent Technologies, Rev. B.06.01).

Samples were injected onto an Agilent AdvanceBio Peptide Mapping UHPLC column (2.7 μm, 150 × 2.1 mm, Agilent Technologies), maintained at 55°C, with a flow rate of 0.4 mL/min. The gradient program started with 2% of buffer B (buffer B: water/acetonitrile/formic acid, 10:89.9:0.1) in the isocratic mode for 5 minutes, then increased linearly to 45% B over 40 minutes, further increased to 95% B over 15 minutes, and remained constant for 5 minutes. After this 65-minute run, the system reverted to the initial conditions (2% of B) for 5 minutes to prepare the column for the next run.

The mass spectrometer operated in positive mode, with a nebulizer gas pressure of 35 psi, drying gas flow of 10 L/min at 250°C, and sheath gas flow of 12 L/min at 300°C. The capillary spray voltage was set to 3500 V, the fragment or voltage to 340 V, and the octupole RF Vpp voltage to 750 V. Profile data was collected for both MS and MS/MS scans in extended dynamic range mode. The MS and MS/MS mass range was 50–1700 m/z, with scan rates of 8 spectra/s for MS and 3 spectra/s for MS/MS. Auto MS/MS mode was used with precursor selection by abundance, allowing a maximum of 20 precursors per cycle. A ramped collision energy was applied with a slope of 3.6 and an offset of −4.8. The same ion was excluded after two consecutive scans.

#### Data processing.

Data processing and analysis were performed using the Spectrum Mill MS Proteomics Workbench (Rev B.04.01, Agilent Technologies). In summary, raw data were extracted under the default settings as follows: non-fixed or variable modifications were selected; [MH] + 50–10000 m/z; a maximum precursor charge of +5; retention time and m/z tolerance of 60 seconds; a minimum signal-to-noise (S/N) ratio for MS of 25; and the identification of 12C signals. The MS/MS search was conducted against the relevant and updated protein database (in this case, Uniprot/Horse) using the following parameters: non-fixed modifications were chosen, and as a variable modification, carbamidomethylated cysteines and tryptic digestion with up to five missed cleavages were included. The ESI-QTOF instrument was selected with a minimum matched peak intensity of 50%, a maximum ambiguous precursor charge of +5, monoisotopic masses, a peptide precursor mass tolerance of 20 ppm, a product ion mass tolerance of 50 ppm, and the calculation of reversed database scores. The autovalidation strategy employed the auto-threshold method to optimize the peptide score for a 1.2% target FDR. Finally, validated results were restricted by a new maximum target protein FDR of 0%.

### Bioinformatic analysis of proteomic data

#### Variance filtering and PCA.

Data were normalized and log2-transformed using Qlucore Omics Explorer (https://qlucore.com). Variables with low overall variance were filtered out, and principal component analysis (PCA) was employed to visualize the dataset in three dimensions. This process helped reduce noise and ensured that the remaining variables were centered and scaled to zero mean and unit variance. The optimal filtering threshold was determined using the projection score. Endometrial fluid proteins with the highest significant difference between healthy mares and jennies were identified using Qlucore Omics Explorer Ver 3.7 (https://qlucore.com). This software works by fitting a linear model for each variable with condition proteins of the uterine samples. P-values were adjusted for multiple testing using the Benjamini-Hochberg method, and variables with adjusted P-values below 0.1 were used for further analysis [49].

#### Enrichment analysis of pathways in the uterine proteome.

Enrichment analysis was performed using the g:Profiler web server (https://biit.cs.ut.ee/gprofiler/gost) [50] using differentially expressed proteins in mares and jennies. The significance of protein list representation was queried against the equine proteome database using a false discovery rate (FDR) threshold of < 0.05 and Fisher’s exact test. Only annotated genes were included, and the Benjamini–Hochberg FDR correction was set at p < 0.05. Subsequently, protein enrichment analysis using human orthologs was performed with g: Profiler web server, which identified key molecular functions, biological processes, and cellular components associated with the differentially expressed proteins. This allowed for the identification of relevant biological terms that may play a role in uterine physiology.

Additionally, the Metascape platform (https://metascape.org) was employed to conduct complementary enrichment analysis using human orthologs [51]. This analysis focused on identifying specific terms related to immune processes, highlighting significant pathways such as “Immune system process (Pathway),” “Regulation of immune system process (Pathway),” and “Negative regulation of immune system process (Pathway).” The enriched pathways offer insights into the role of uterine proteins in the regulation of the immune system and their potential impact on equine reproductive physiology.

## Results

### Endometrial cytological and bacteriological (culture-dependent processing) results

The mares and jennies included in the study were negative for endometritis on cytological and bacteriological analyses.

**16S rRNA analysis reveals similar microbiota profiles in mares and jennies with slight variations in the richness and dominance of some community members.**

**Fig 1:**
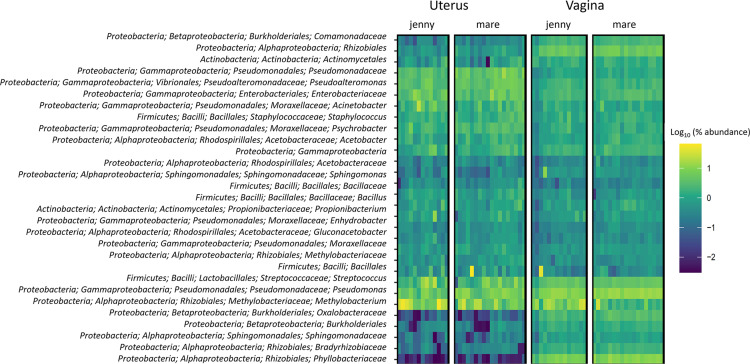
Heatmap representing the most abundant features from the vaginal and/or uterus microbiota in jennies and mares. The normalized features abundances of all OTUs with a minimum frequency of 1,000 is represented as the log10 percent abundance, following the color-coding shown in the legend.

The heatmap displays the most relative abundant ASVs from the vaginal and uterine microbiota in jennies and mares globally, highlighting the similarities between microbial communities, despite minor differences mainly corresponding to individual variations (Fig 1).

### Differences in the vaginal microbiota between mares and jennies

**Fig 2:**
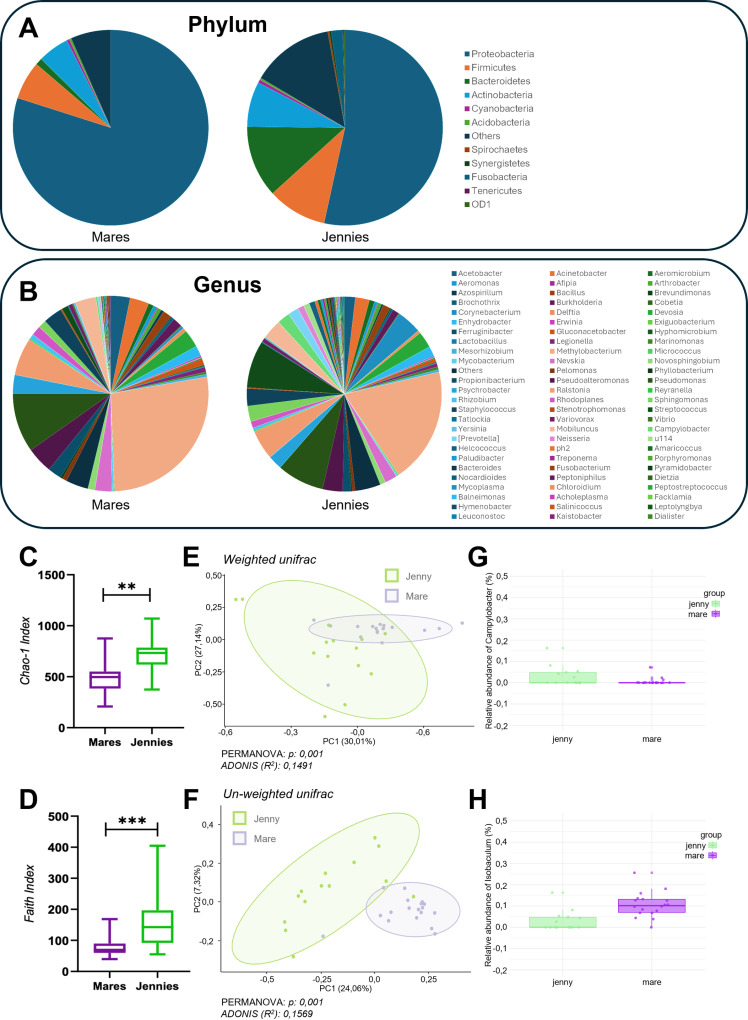
A,B: The pie chart shows the relative abundances (%) of the identified taxa in the vaginal microbiota of mares and donkeys at the phylum (A) and genus levels (B). C,D (Alpha diversity analysis): The box plot (depicting minimum and maximum values) illustrates alpha diversity measures of the vaginal microbiota in mares and jennies, including the Chao-1 index (C) and the Faith’s Phylogenetic Diversity index (D). Significant differences between the two species are highlighted (C: **p = 0.0026; D: ***p = 0.0002)”. E,F (Beta diversity analysis): PCoA plots (Principal coordinate analysis: PCoA) representing the beta diversity of the vaginal microbiota of mares (purple) and jennies (green). Weighted UniFrac distance matrix is represented in fig D and Un-weighted in fig F. Statistical analyses were performed using PERMANOVA (p = 0.001) to assess the differences in community composition and ADONIS to evaluate the effects of explanatory variables on the variation in the data. G,H: ANCOM-BC analysis identified differences in only two taxa between the vaginal microbiota of mares and jennies. The bar plot illustrates the two genera that were differentially abundant: Campylobacter, which is more prevalent in jennies (G), and Isobaculum, which is more prevalent in mares (H).

The total number of features obtained after performing the quality steps in the samples taken from the vagina of mares and jennies (n= 32) were 7,528 with a mean of 29,823 features per sample (range 5,853–103,539). The taxonomy assignment revealed high similarities between species. The most abundant phylum identified in the vaginal microbiota of both animal species were Proteobacteria (79.92 vs 53.44%), Firmicutes (6.33 vs 9.83%), Actinobacteria (5.15 vs 7.48%), and Bacteroidetes (1.16 vs 11.88%) (Fig 2A). In jennies, the microbial profile at the phylum level was similar to that of mares, though Bacteroidetes had a higher relative abundance than Firmicutes. At the level of genus, *Methylobacterium, Pseudomonas, Pseudoalteromonas, Pseudoxanthomonas, Acetobacter, Acinetobacter, Staphylococcus, Psychrobacter, Propionibacterium*, *Bacillus, Sphingomonas, and Streptococcus* were the genera with a high relative abundance in the vagina of both species (Fig 2B). The genus *Lactobacillus* had a low relative abundance or was absent in the vagina of most of the animals independent of the species (mares: 0.17% and jennies: 0.1%) (S1 Table).

Alpha and beta diversity were also explored in the vagina of these two species. The vaginal microbiota in jennies exhibited higher richness (Chao-1; p = *0.0026*) and diversity (Faith-PD, p = *0.0002*) than in mares (Fig 2CD). Beta diversity analysis using weighted and unweighted Unifrac distance metrics revealed significant differences between vaginal microbiota of both species (*p < 0.001*) (Fig 2EF). However, differential analysis performed using ANCOM-BC collapsed at genus level, revealed differences in only two taxa: *Campylobacter*, with higher relative abundance in jennies, and *Isobaculum* more relative abundant in mares, as represented in Fig 2GH.

### Differences in the uterine microbiota between mares and jennies

**Fig 3:**
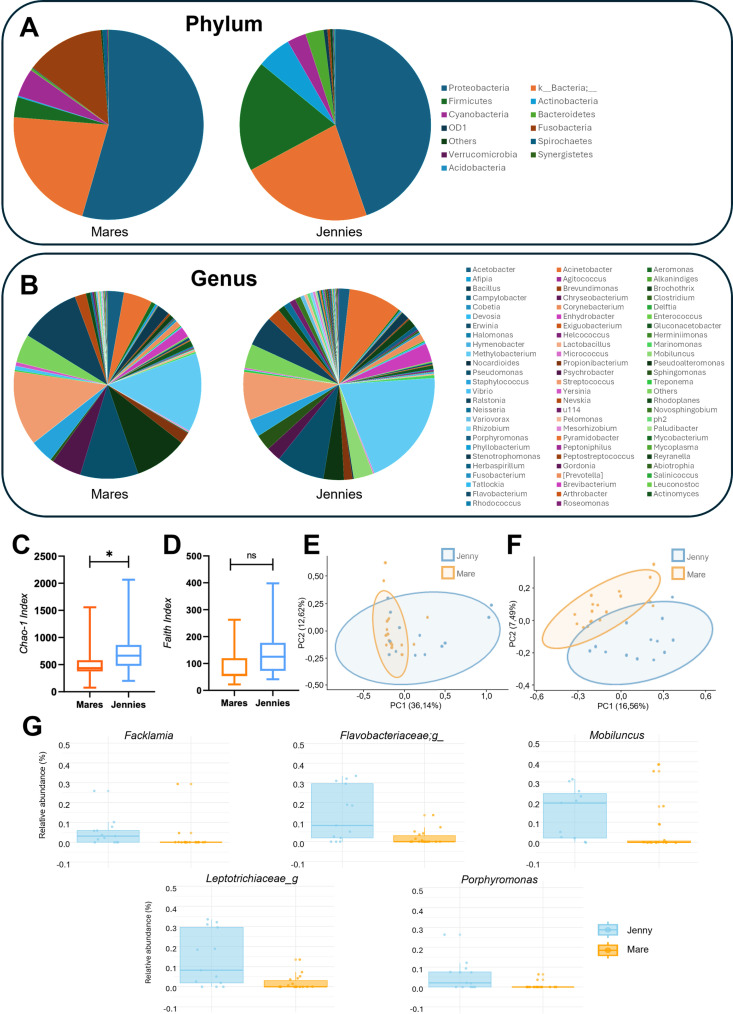
A,B: The pie chart shows the relative abundances (%) of the identified taxa in the uterine microbiota of mares and donkeys at phylum (A) and genus levels (B). C,D (Alpha diversity analysis): The box plot (depicting minimum and maximum values) illustrates alpha diversity measures of the uterine microbiota in mares and jennies, including the Chao-1 index (C) and the Faith’s Phylogenetic Diversity index (D). Significant differences between the two species were only identified in chao-1 index (C): *p = 0.0411). E,F (Beta diversity analysis): PCoA plots (Principal coordinate analysis: PCoA) representing the beta diversity of the uterine microbiota of mares (orange) and jennies (blue). Weighted UniFrac distance matrix is represented in fig D and Un-weighted in fig F. Statistical analyses were performed using PERMANOVA (p = 0.001) to assess the differences in community composition and ADONIS to evaluate the effects of explanatory variables on the variation in the data. G: The ANCOM-BC analysis identified significant differences in the relative abundance (%) of five taxa found in the uterus of mares and jennies. The bar plot shows the genera that were significantly more abundant in jennies compared to mares.

The samples taken from the uterus of jennies and mares (n=31) were submitted to 16S rRNA sequencing to reveal the microbiome composition. The total number of features found was 11,584, with a mean of 20,498 features per sample (range 1,263–68,909). The taxonomy assignment revealed high similarities between species. The main phyla found in the uterus of mares and jennies were Proteobacteria (54.4 vs 44.56%) followed by Firmicutes (21.78 vs 18.74%), Bacteroidetes (4.80 vs 3.06%), and Actinobacteria (3.45 vs 5.78%) (Fig 3A). The most abundant features identified in the uterus location were *Methylobacterium* (13.60 vs. 19.93%), *Streptococcus* (12.92 vs 8.10%), *Pseudoxanthomonas* (10.55 vs. 5.13%), *Pseudomonas* (9.99 vs. 8.12%), *Pseudoalteromonas* (9.14 vs 3.44%), *Psychrobacter* (5.16 vs 2.46%), *Acinetobacter* (4.95 vs.8.89%), *Staphylococcus* (4.04 vs 2.99%), *Bacillus* (2.17 vs. 1.39%), *Sphingomonas* (0.5 vs. 2.70%) and *Corynebacterium* (0.99 vs 1.29%) (Fig 3B). The relative abundance of *Lactobacillus* was 0.36% in mares and 0.22% in jennies (S1 Table).

Alpha analysis revealed that the uterine microbiota in jennies exhibited higher richness (Chao 1; *p < 0.05*) than in mares, while analysis including the phylogenetic relationships (Faith index) did not show significant differences (Fig 3CD). Principal coordinate analysis (PCoA) was carried out using weighted and unweighted UniFrac distance metrics. Uterine samples from mares and jennies were grouped into two distinct clusters in both quantitative (weighted UniFrac) and qualitative (unweighted UniFrac) analyses (PERMANOVA, p = 0.001 and p = 0.002, respectively) (Fig 3EF). Since both animal species shared a high number of the most abundant taxa, these results suggest that the differences observed are primarily driven by the low abundant taxa. Consistently, the quantitative approach showed a higher effect size (R²=13.27%) compared to the qualitative approach (R²= 8.89%) (Fig 3EF).

Differential abundance analysis of the uterine microbiota between mares and jennies was conducted using ANCOM-BC, with feature tables collapsed at the genus level. The analysis identified five taxa with significant differences in abundance between the uteri of both animal species. These included two unclassified genera from the Leptotrichiaceae and Flavobacteriaceae families, as well as *Porphyromonas, Mobiluncus*, and *Facklamia.* In all cases, these genera were found to be more abundant in the uterus of jennies compared to mares (Fig 3G).

### Metagenome prediction and functional inference of the uterine microbiota

**Fig 4:**
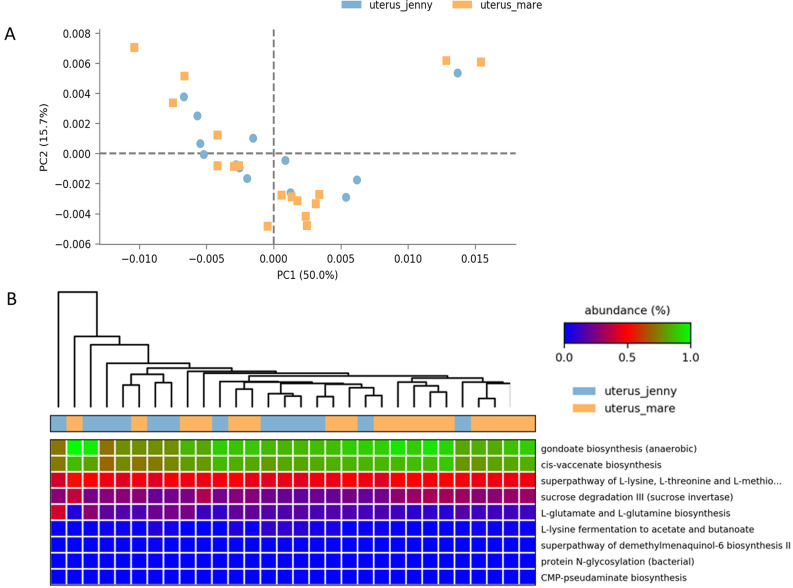
A. PCA plots based on metagenomic predictions show no distinct clustering of uterine microbiota across different animal species (jennies: blue, mares: orange). B: Predicted KEGG functional pathways using PICRUSt analysis. The heat map shows the most statistically significant differential pathways between the uterine microbiota of mares (Orange) and jennies (blue) (Kruskal–Wallis post-hoc test with Bonferroni correction, *p* value < 0.05; effect size < 0.8). B: The scatter plots illustrated the high paired correlation observed between predicted functional pathways of both species (R^2^= 0.997).

To investigate whether the observed compositional differences might have functional implications, the predicted functional pathways were performed based on 16S rRNA sequences for the uterine microbiomes of both species. The similarities between the predicted functional pathways in the uterus of both animal species was evident in the PCA and the high paired correlation observed (R^2^= 0.997) (Fig 4A).

Differential analysis identified only nine altered low-abundance pathways between the uteri of both species (Kruskal-Wallis with Bonferroni correction, p < 0.05; effect size < 0.8). Among these, only the gondoate and cis-vaccenate biosynthetic pathways had relative abundances of approximately 1% (Fig 4B).

### Differences between vaginal and uterine microbiota (niche) within each animal species

**Fig 5:**
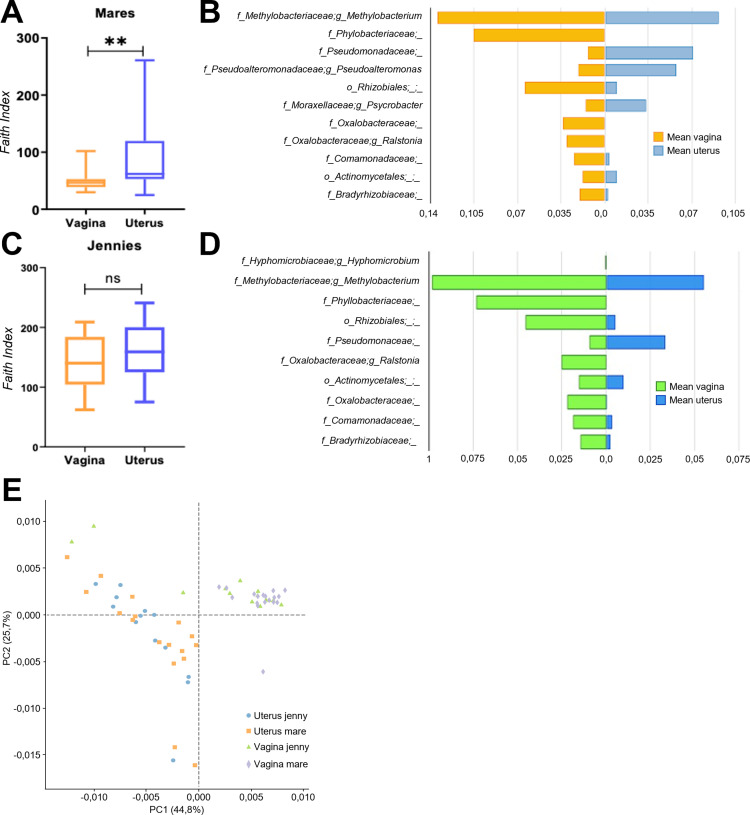
A. Box plot showing Faith’s Phylogenetic Diversity index for vaginal and uterine microbiota in mares, highlighting significant differences between the two niches (**p < 0.001). B: The mean relative abundance of genera identified as differentially abundant between the uterus and vagina in mares. The top 10 genera, as detected by ANCOM-BC, are illustrated. C: Box plot showing Faith’s Phylogenetic Diversity index for vaginal and uterine microbiota in jennies. D: The mean relative abundance of genera identified as differentially abundant between the uterus and vagina in jennies. The top 10 genera, as detected by ANCOM-BC, are illustrated. E: PCA analysis reveals clear clustering by niche (but not by animal species), indicating that the differences in metabolic pathways are primarily driven by the niche.

Alpha diversity analysis revealed that the uterine microbiome of the mare exhibited greater bacterial diversity (though not richness) compared to the vaginal microbiota (Faith index: 92.07 ± 64.67 vs. 49.24 ± 16.38; *p < 0.001*) (Fig 5A). A similar trend was observed in jennies, although the differences were not statistically significant (Fig 5C).

In the PCoA plot based on weighted and unweighted UniFrac distances (beta diversity), the vaginal and uterine microbiota were grouped into two distinct clusters in mares (PERMANOVA, p = 0.001 for both weighted and unweighted analysis) and in jennies (PERMANOVA, p = 0.016 and p = 0.002 for unweighted and weighted analysis, respectively). According to the Adonis function, the niche explained 23.87% of the variations in the microbiota of mares and 13.62% of the differences in jennies using weighted UniFrac distance matrices, highlighting the differences that exist in these two niches regardless of the animal species.

To unravel the differences in bacterial communities inhabiting the different niches, we performed analysis of the composition of the microbiome with bias correction (ANCOM-BC). When comparing the vagina and uterus of mares, 30 ASVs were detected as differentially abundant at family level, while 60 ASVS were detected differentially at genera level. Top 10 most globally abundant genera detected by ANCOM-BC are depicted in Fig 5B. The full list of the detected genera is included in S2 Table. Most of the identified taxa in both locations were less abundant in the uterus than in the vagina. However, the Pseudomonadaceae family, and *Pseudoalteromonas,* and *Psychrobacter* genera were found among the identified features more relatively abundant in the uterus of mares (Fig 5B).

Differential analysis between the vagina and uterus of jennies identified 53 taxa with significant differences in abundance (S2 Table). Of these, 39 taxa were present in the uterus but at lower abundances compared to the vagina, with the exception of Pseudomonacea family, which was more abundant in the uterus. Fig 5D illustrates the top 10 genera identified as differentially abundant between the uterus and vagina in donkeys, as determined by ANCOM-BC.

### Metagenome prediction and functional analysis inference

PCA of the functional pathways showed a clear clustering of the samples by niche in mares and in jennies, indicating that the differences detected at the compositional level (beta diversity) are also found when predicting the metabolic pathways involved (Fig 5E). However, no differential clustering was observed by animal species, indicating that the differences in metabolic pathways detected are mainly driven by niche and not by animal species. In agreement, a higher correlation within animal species in the paired comparisons supports this finding (R^2^= 0.94 uterus vs. vagina mare < R^2^ = 0.959 uterus vs. vagina jenny < R^2^ = 0.993 vagina mare vs. vagina jenny < R^2^ = 0.997 uterus jenny vs. uterus mare). The main metabolic pathways in the uterus and vagina of both species are shown in S3 Table. Among the primary metabolic pathways identified in the uterus of both species were: pyruvate fermentation to isobutanol (PWY-7111), gondoate biosynthesis (anaerobic) (PWY-7663), cis-vaccenate biosynthesis (PWY-5973), L-isoleucine biosynthesis II (PWY-5101), CDP-diacylglycerol biosynthesis I and II (PWY-5667, PWY-1319), L-valine biosynthesis (VALSYN-PWY), fatty acid elongation – saturated (FASYN-ELONG-PWY) and pentose phosphate pathway (non-oxidative branch) (NONOXIPENT-PWY).

### Differences in the uterine proteome between mares and jennies

**Fig 6:**
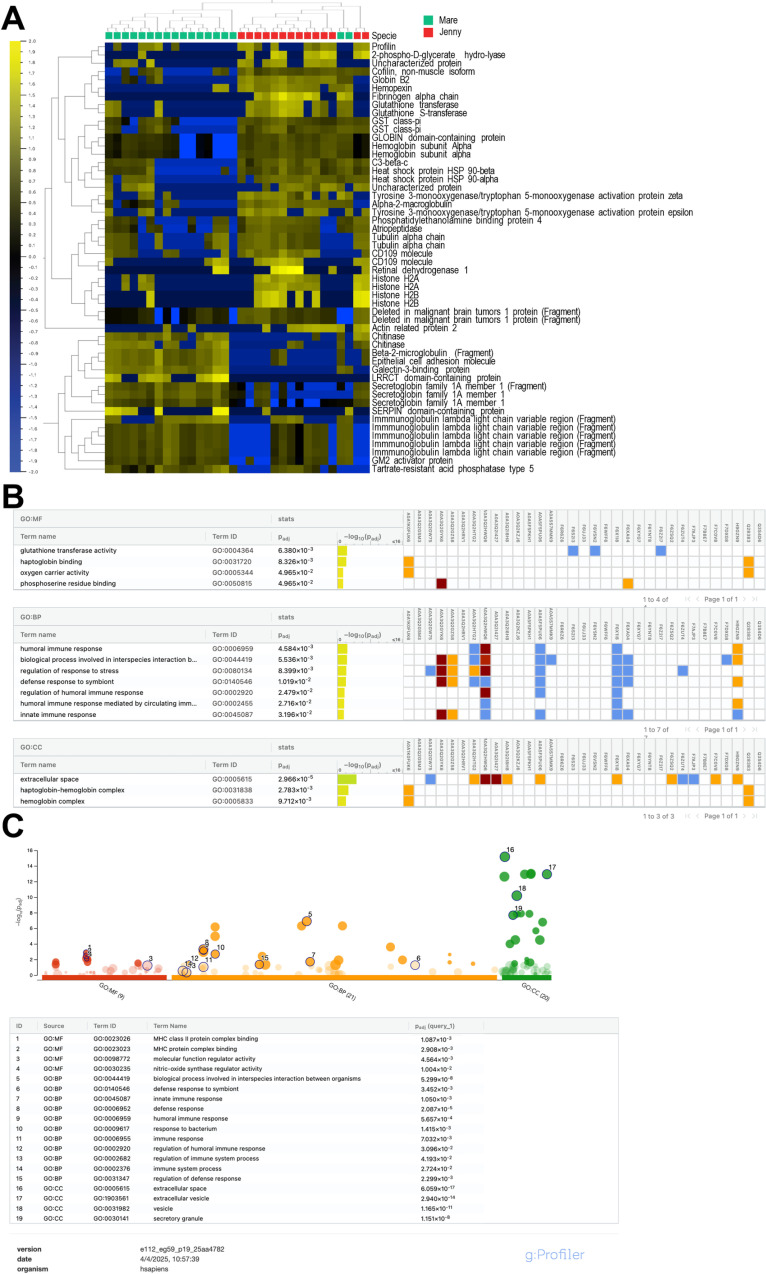
A. Heatmap showing the proteins differentially expressed in the endometrial proteome of mares and jennies. Proteins are classified following hierarchical clustering. Samples from mares correspond to the green marks, and samples from jennies correspond to the red marks. The heat map code is present, with yellow areas representing larger amounts of protein and blue areas smaller amounts of protein. Proteins were normalized, filtered by a fold change of 3.6 with p = 0.001 and q = 0.004. B: The table generated from the g:profiler enrichment analysis using the equine proteome displays the Gene Ontology (GO) categories in which the differentially expressed proteins in mares and jennies are involved. These categories include Molecular Function (MF), Biological Processes (BP), and Cellular Component (CC). The most representative proteins are shown. For each main category, the table lists the term name, term ID, adjusted p-values, and the proteins associated with each category, identified by their serial number. Notably, seven proteins are associated with the defense response to symbionts. C: Manhattan plots obtained from g:profiler enrichment analysis using human orthologs highlight the proteins associated with various GO terms: red represents Molecular Function, yellow indicates Biological Process, and green corresponds to Cellular Component. The p-values are plotted on the y-axis, with further details provided in the results table below the image.

The heatmap displays the common proteins differentially abundant in the uterine proteome of mares and jennies (Fig 6A). We identified a total of 52 significantly differentially expressed uterine proteins between mares and jennies, 17 upregulated proteins in mares, and 35 upregulated in the uterus of jennies.

### GO enrichment analyses of uterine proteins differentially expressed

GO enrichment analyses of differentially expressed uterine proteins, conducted using g:Profiler, categorized into three GO classes: molecular function (MF), cellular component (CC), and biological process (BP). The common uterine proteins primarily participate in biological processes related to immune response, including humoral immune response (GO:0006959), humoral immune response mediated by circulating immunoglobulin (GO:0002455), defense response to symbiont (GO:0140546), or innate immune response (GO:0045087) among others (Fig 6B). Seven proteins were associated to the defense response to symbiont: C3-beta-c (A0A3Q2HWQ6), Immunoglobulin heavy constant mu (H9GZN9), heat shock protein HSP 90-alpha (A0A3Q2GZ58), Tyrosine 3-monooxygenase/tryptophan 5-monooxygenase activation protein zeta (A0A3Q2GYK6), Fibrinogen alpha chain (A0A3Q2HTG2), Alpha-2-macroglobulin (A0A5F5PU06), Hemopexin (F6X1I8), and Tyrosine 3-monooxygenase/tryptophan 5-monooxygenase activation protein epsilon (F6XA04). Additionally, other overexpressed proteins with immunoregulatory functions were identified, such as the CD109 molecule (F6V1V8) and the SERPIN domain-containing protein (F7C0V8).

Furthermore, we performed protein enrichment analysis using human orthologs and identified molecular functions such as MHC class II protein complex binding (GO:0023026), or molecular function regulator activity (GO:0098772), and nitric-oxide synthase regulator activity (GO:0030235). In terms of biological function, we observed enrichment in processes involved in interspecies interaction between organisms (GO:0044419), humoral and innate immune response, and once again defense response to symbiont. Several of the proteins identified as part of the cellular component were found to be associated with extracellular exosomes, secretory vesicles, and other extracellular vesicles (Fig 6C).

**Fig 7:**
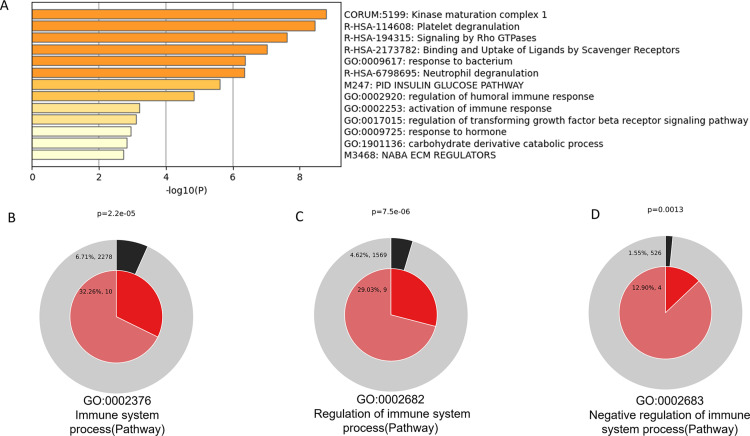
A: shows all statistically enriched terms (including GO/KEGG terms, canonical pathways, hallmark gene sets, among others) based on the default options of Express Analysis in Metascape. Accumulative hypergeometric p-values and enrichment factors were calculated and used for filtering the terms. The remaining significant terms were hierarchically clustered into a tree based on Kappa-statistical similarities among shared genes. A Kappa score threshold of 0.3 was applied to form term clusters. Among the most significant GO terms are GO:0009617 (response to bacterium), GO:0002920 (regulation of humoral immune response), GO:0002253 (activation of immune response), GO:0017015 (regulation of transforming growth factor beta receptor signaling pathway), and GO:0009725 (response to hormone). 7 B-D Enrichment of genes matching membership term: Immune system process (pathway), Regulation of immune system process (pathway) and negative regulation of immune system process (pathway). The outer pie shows the number and the percentage of genes in the background proteome that are associated with the membership (in black); the inner pie shows the number and the percentage of genes in the individual input gene list that are associated with the membership. B: Immune system Process (p = 2.2e-05). C: Regulation of Immune system process (p = 7.5e-06). D: Negative regulation of Immune system process (p = 0.0013).

Gene enrichment analysis, conducted using the Metascape platform and orthologous human ENSG genes, identified several enriched Gene Ontology (GO) terms based on the default choices under Express Analysis. Notably, these included GO:0009617 (response to bacterium), GO:0002920 (regulation of humoral immune response), activation of immune response (GO: 0002253) and GO:0017015 (regulation of transforming growth factor beta receptor signaling pathway), among others (Fig 7A).

Metascape platform was also used to search for the enrichment of specific terms: “Immune system process (Pathway),” “Regulation of immune system process (Pathway),” and “Negative regulation of immune system process (Pathway)” (Fig 7B-D). The outer pie chart illustrates the number and percentage of genes in the background (whole proteome) associated with the membership (in black); the inner pie shows the number and percentage of genes related to the membership (uterine proteome). The p-value indicates that the term is significantly enriched in the uterine proteome (Fig 7B-D).

## Discussion

This study reveals that the reproductive tract of healthy mares and donkey jennies share similar microbiota and functional pathways for the first time. Our results indicate that the vaginal microbiome of mares is predominantly characterized by the phylum Proteobacteria, followed by Firmicutes, Actinobacteria, and Bacteroidetes. This profile is consistent with previous equine studies, although there were minor differences in relative bacterial abundance that may be attributed to variations in sampling location within the vagina [1,18]. The samples obtained from our study were taken from the cranial vagina surrounding the cervix, while the other studies collected samples from the caudal vaginal wall or vestibule [18]. This highlights the importance of sampling location in microbiome research and its potential impact on outcomes. The vaginal microbiome of jennies displayed a higher bacterial richness (Chao-1) and diversity (Faith's PD) compared to the mare. This difference could be due to the anatomical features of the vulva and vagina in this species. The vulva of jennies is positioned entirely below the pelvic floor, with a more inclined vaginal vestibule than in mares [52], which may lead to increased exposure of the vaginal microbiota to external factors, thereby promoting greater microbial diversity.

Previous studies have examined the existence of lactic acid bacteria and lactobacilli in mares’ vaginas using traditional culture techniques [53]. Certain strains of Lactobacillus have been utilized as probiotics to modulate the vaginal and uterine microbiota in women, preventing, and treating reproductive diseases [54]. Recently, *Holyoak et al*. found that Lactobacillus was the third most abundant genus in the endometrial microbiome of healthy mares of different geographical locations, with a relative abundance of 7.5% [24]. While our findings are similar in terms of taxonomy, the relative abundance of *Lactobacillus* in our vaginal and uterine samples was notably lower or even absent, in both species. These results are consistent with recent research in jennies, which identified Lactobacillus in the vaginal microbiome as the taxon most associated with endometritis in jennies [1]. Instead, we found that *Streptococci* (another lactic acid bacteria used as potential probiotics) are key components of the core microbiota in both the vagina and uterus of both species. Strains like *Streptococcus infantarius, Streptococcus thoraltensis, and Streptococcus zooepidemicu*s are resident vaginal bacteria in healthy mares [21]. However, several beta-hemolytic species of Streptococcus, including S. equi subsp. zooepidemicus, S. dysgalactiae subsp. equisimilis, and S. equi subsp. equi, are frequently isolated from the uteri of mares with infectious endometritis [55,56]. These pathogenic species can infiltrate and persist within the uterine glands [57,58] or inside the endometrial cells [59] of chronically infected mares, remaining dormant and evading standard culture methods, but may reactivate and cause acute infectious endometritis [58]. We were unable to identify the specific species within the *Streptococcus* genus present in the commensal microbiota of the animals examined due to the limitations of the sequencing technique used. While 16S rRNA sequencing is a widely utilized method for microbial profiling, it often lacks the resolution required for species-level identification, typically providing results at the family or genus level. However, since the samples for sequencing were obtained from uterine lavage of healthy animals, the bacteria identified were likely on the surface of the endometrial epithelium rather than within deeper tissues, making it unlikely that they were latent intracellular pathogens. Further research is needed to identify specific *Streptococcus* species in the healthy uterine microbiome and explore their potential as probiotics for infection prevention and microbial balance.

Bacterial endometritis is a well-recognized condition resulting from a failure in uterine defense mechanisms, allowing the proliferation of pathogenic or opportunistic bacteria [60,61]. Historically, the uterus was considered a sterile environment, and bacterial endometritis has been primarily attributed to the invasion and growth of such organisms during mating or their ascension from the lower genital tract [62]. The most common uterine bacteria related to endometritis in mares included *S. equi subsp. zooepidemicus, E. Coli, Pseudomonas aeruginosa or Staphylococus aureus [*63,64]. Notably, genera such as *Streptococcus, Pseudomonas, Bacillus, Staphylococcus, and Corynebacterium* were identified as abundant members of the uterine commensal microbiota in both species analyzed in our study. These findings suggest that commensal bacteria likely play a protective role in maintaining the stability of the complex microbial environment by preventing pathogen overgrowth and latent microbe activation. Although further studies are needed to isolate and fully characterize the identified commensal bacteria, this new understanding highlights the critical importance of maintaining microbial equilibrium within the uterus.

Previous research has unveiled that the composition of the microbiome differs depending on the anatomical location [7,19,20]. The present study revealed that while the uterine and vaginal microbiome in mares and donkeys shares many bacterial taxa, there are significant differences in diversity, composition, and functional pathways between the two niches. Indeed, our results indicate that the differences observed in functional analysis are primarily driven by the specific niches, rather than by animal species.

Consistent with previous studies [1,24], the most abundant phyla in the uterus of mares and jennies were Proteobacteria, followed by Firmicutes, Bacteroidetes, and Actinobacteria. Despite taxonomic similarities, beta diversity analysis revealed distinct clustering between animal species. These differences were likely due to variations in low-abundance taxa, as only five genera showed higher proportions in the uterus of jennies compared to mares, each with a relative abundance of less than 0.3%. However, despite these compositional differences, PICRUSt analysis revealed no higher differences at the functional level within the same niches. These findings suggest that the commensal organisms inhabiting the reproductive tract of both mares and jennies share identical functional pathways. The identification of microbial metabolic pathways is often more consistent across individuals compared to the detection of specific bacterial species due to the functional redundancy of bacteria [65]. This consistency makes them potentially more reliable indicators of physiological or pathological states.

When we predicted the functional capacities of the uterine microbiota, we identified two upregulated anti-inflammatory microbial metabolic pathways: pyruvate fermentation to isobutanol (PWY-7111), a key precursor in the production of short-chain fatty acids (SCFAs) and gondoate biosynthesis (PWY-7663). SCFAs, including acetate, propionate and butyrate are primary end metabolic products of microbial fermentation and play a crucial role in maintaining gut health [66]. In gut microbiota, SCFAs induce tolerogenic and anti-inflammatory enterocyte phenotypes. Moreover, SCFAs induce downregulation of pro-inflammatory innate immune cells and their cytokines, reducing inflammation [67–69] and promoting the upregulation of anti-inflammatory T regulatory (Treg) cells [70,71]. One of the pathways that showed slight differences between mares and donkeys was L-lysine fermentation to acetate and butanoate (P163-PWY, p < 0.041), two SCFAs that contribute to immune homeostasis [66]. Some bacterial genera known to ferment L-lysine to acetate and butanoate are *Clostridium, Fusobacterium*, and *Porphyromonas*. The genera *Fusobacterium* and *Porphyromonas* were present in both species, with a higher relative abundance in jennies than in mares. Another of the enriched pathways identified in the uterus of both species was gondoate biosynthesis. Interestingly, a study on Crohn's disease observed a decrease in gondoate biosynthesis pathway during periods of disease exacerbation (proinflammatory state) [72]. This finding suggests that the presence of the gondoate biosynthesis pathway in the uterine microbiota may play a significant role in maintaining a balanced state and promoting uterine health.

Extensive evidence shows that commensal microbes regulate both innate and adaptive immune responses to maintain immune homeostasis in the intestinal tract [73]. However, to the best of our knowledge, no studies have investigated the cellular and molecular interactions between uterine microbes and the immune system. The enrichment analysis of the uterine proteome revealed that numerous overexpressed proteins in both species were associated with adaptive and innate immune responses, their regulation, and defense responses to symbionts. Particularly, seven of the differentially expressed proteins were related to responses to symbionts. Some of these proteins, such as C3 beta or Immunoglobulin heavy constant mu, are components of the complement system. Immunoglobulin heavy constant mu (H9GZN9) is a component of IgM that plays a key role in the classical complement pathway. Secretory immunoglobulins (sIgs), particularly IgA, are key adaptive immune molecules that maintain mucosal homeostasis by neutralizing pathogens and supporting commensal microbiota colonization. Recent findings suggest that IgM may contribute to maintaining gut microbiota symbiosis [74]. While the role of uterine-secreted IgM remains largely unexplored, the overexpression of IgM components in uterine fluid suggests a likely role in maintaining microbial balance in the equine uterus, as it is associated with the response to symbionts. Consistent with our findings, previous research also reported overexpression of C3b in the uterine flush fluid of healthy mares compared to those with endometritis, indicating that reduced C3b levels in infected mares may result from decreased local production or increased C3b loss during infection or inflammation [27]. The animals in our study were healthy females, ensuring that there were no other stimuli except for their own microbiota. Furthermore, these findings suggest that the commensal microbiota plays a vital role in regulating the complement system, preventing excessive activation during a balanced state, and ensuring it targets only true pathogens.

Gene enrichment analysis, conducted using orthologous human ENSG genes, revealed that the enriched genes are involved in the host's response to bacterial presence and the regulation of the transforming growth factor-beta (TGF-beta) receptor signaling pathway. This pathway plays a crucial role in the regulation of immune tolerance, as TGF-beta is essential for the differentiation and function of regulatory T cells (Tregs) [75]. Tregs express high levels of TGF-beta, which they use to suppress the activation of other T cells and help prevent exaggerated immune responses [75,76]. One key regulator of this pathway is CD109, a protein that binds to TGF-beta and inhibits its signaling [77]. Our study found an overexpression of two CD109 molecules (F6V1V8 and A0A3Q2GW75) in the uterus of healthy mares and especially jennies, suggesting that this pathway may play a significant role in regulating immune system interactions with the uterine commensal microbiota.

Finally, when we conducted protein enrichment analysis with human orthologs, we observed that many of the identified proteins were associated with molecular functions such as MHC class II protein complex binding (GO:0023026) and nitric-oxide synthase (NOS) regulator activity (GO:0030235). MHC class II complex proteins are crucial for antigen presentation by antigen-presenting cells (APCs). These proteins may modulate local immunity by presenting commensal microbial antigens, promoting immune tolerance toward the uterine microbiota. Nitric oxide synthase (NOS) regulates local immune responses through the immunomodulatory effects of nitric oxide (NO) [78]. The activity of NOS can be influenced by various factors, including the presence of certain microbial metabolites. Enrichment in nitric-oxide synthase regulator activity might reflect an adaptive mechanism by which the uterine microbiota interacts with the immune system. This could help enhance immune tolerance and regulate inflammation, which is essential for maintaining the immune system balanced and providing effective protection against pathogens, all while supporting beneficial microorganisms.

Furthermore, all these findings indicate that the uterine microbiota may play a vital role in maintaining immune balance.

## Conclusion

This study provides a comprehensive multi-omic analysis of the microbiota in the reproductive tracts of healthy mares and jennies, unveiling that both species exhibit high similarities in microbiota profiles and uterine-predicted metabolic pathways, yet notable differences in diversity and composition influenced by niche-specific factors.

The enrichment analysis of the uterine proteome revealed that numerous overexpressed proteins in both species were associated with adaptive and innate immune responses, their regulation, and defense responses to symbionts. These results suggested that the equine uterus harbors a unique low-biomass microbiota that may influence local immune responses by modulating inflammation and promoting tolerance to commensal microorganisms. Considering the limitations of the present study regarding the number of samples and the inherent constraints of the sequencing techniques used, further investigation is needed to confirm the interaction between the microbiota and the uterine immune system, which could lead to novel therapeutic approaches for improving uterine health and fertility.

## Supporting information

S1 Table Raw data of OTUs (Relative abundance of study samples at the genus and phylum levels). Samples were collected from mares and jennies, including vaginal samples obtained with swabs and uterine samples obtained from uterine fluid during two consecutive estrous cycles, 1 and 2. (XLSX)

S2 Table The full dataset obtained from the microbiome composition analysis with bias correction (ANCOM-BC). The analysis was conducted to compare differences between niches (vagina and uterus within the same species). In mares, a total of 30 ASVs were identified as differentially abundant at the family level when comparing the vaginal and uterine microbiomes, while 60 ASVs showed significant differences at the genus level. In jennies, differential analysis between the vaginal and uterine microbiomes identified 53 taxa with significant abundance differences. (XLSX)

S3 Table Differential analysis of metabolic pathways in the uterus and vagina of both species. Statistical analysis was performed using the Kruskal-Wallis test with Bonferroni correction (p < 0.05; effect size < 0.8). (XLSX)
